# IL-6-Driven pSTAT1 Response Is Linked to T Cell Features Implicated in Early Immune Dysregulation

**DOI:** 10.3389/fimmu.2022.935394

**Published:** 2022-07-13

**Authors:** Katharina Lambert, Kirsten E. Diggins, Britta E. Jones, Christian Hundhausen, Megan D. Maerz, Anne M. Hocking, Srinath Sanda, Carla J. Greenbaum, Peter S. Linsley, Karen Cerosaletti, Jane H. Buckner

**Affiliations:** ^1^ Center for Translational Immunology, Benaroya Research Institute at Virginia Mason, Seattle, WA, United States; ^2^ Center for Systems Immunology, Benaroya Research Institute at Virginia Mason, Seattle, WA, United States; ^3^ Immune Tolerance Network, Seattle, WA, United States; ^4^ Department of Pediatrics, University of California, San Francisco, CA, United States; ^5^ Center for Interventional Immunology and Diabetes Program, Benaroya Research Institute at Virginia Mason, Seattle, WA, United States

**Keywords:** interleukin-6 (IL-6), interleukin-6 receptor (IL-6R), T cells, STAT1, STAT3, apoptosis, autoimmunity

## Abstract

Elevated levels and enhanced sensing of the pro-inflammatory cytokine interleukin-6 (IL-6) are key features of many autoimmune and inflammatory diseases. To better understand how IL-6 signaling may influence human T cell fate, we investigated the relationships between levels of components of the IL-6R complex, pSTAT responses, and transcriptomic and translational changes in CD4^+^ and CD8^+^ T cell subsets from healthy individuals after exposure to IL-6. Our findings highlight the striking heterogeneity in mbIL-6R and gp130 expression and IL-6-driven pSTAT1/3 responses across T cell subsets. Increased mbIL-6R expression correlated with enhanced signaling *via* pSTAT1 with less impact on pSTAT3, most strikingly in CD4^+^ naïve T cells. Additionally, IL-6 rapidly induced expression of transcription factors and surface receptors expressed by T follicular helper cells and altered expression of markers of apoptosis. Importantly, many of the features associated with the level of mbIL-6R expression on T cells were recapitulated both in the setting of tocilizumab therapy and when comparing donor CD4^+^ T cells harboring the genetic variant, *IL6R* Asp358Ala (rs2228145), known to alter mbIL-6R expression on T cells. Collectively, these findings should be taken into account as we consider the role of IL-6 in disease pathogenesis and translating IL-6 biology into effective therapies for T cell-mediated autoimmune disease.

## Introduction

The cytokine interleukin-6 (IL-6) is a key contributor to T cell dysregulation in the context of autoimmunity, promoting pro-inflammatory T cell lineages, including T helper 17 cells (Th17) and T follicular helper cells (Tfh), inhibiting regulatory T cell (Treg) lineages (1, 2), as well as supporting the proliferative and survival capacities of pathogenic T cells (3, 4). To date, most of this work has been done in murine models of autoimmunity with less known about the T cell response to IL-6 in humans. For example, IL-6 signaling is indispensable in driving Th17 and Tfh-driven pathogenic T cell responses in murine models, both *in vitro* and *in vivo* (5–9). Yet, the contribution of IL-6 in these processes is not clear in human T cells, as IL-6 is dispensable for the differentiation of these subsets *in vitro* (10, 11). However, the importance of IL-6 signaling in driving autoimmune processes in humans is supported by a genetic variant in the *IL6R* locus, rs2228145 Asp>Ala (AA>CC). T cells with the minor CC genotype show impaired classic IL-6 signaling due to enhanced a disintegrin and metalloproteinase (ADAM)-mediated shedding of the membrane-bound IL-6 receptor (mbIL-6R) compared to the AA genotype and are associated with protection from development of rheumatoid arthritis and type 1 diabetes (T1D) (12). Furthermore, blocking the IL-6 pathway with tocilizumab, a monoclonal antibody targeting the IL-6R, shows efficacy (13, 14) and is approved for treatment of rheumatoid arthritis (15), juvenile idiopathic arthritis (16), giant cell arteritis (17), cytokine release syndrome (18), and systemic sclerosis (19), supporting a role for IL-6 signaling in disease pathogenesis.

IL-6 through its receptors IL-6R and the signal transducing beta-receptor subunit gp130 drives a signaling cascade involving phosphorylation of STAT proteins, specifically STAT1 and STAT3 (20). Three modes of signaling in response to IL-6 have been described. In classical IL-6 signaling, IL-6 binds to mbIL-6R and gp130 (21). Trans-signaling depends on IL-6 binding to soluble-IL-6R (sIL-6R) (22) and cluster-signaling involves intracellular loading of IL-6/IL6R complexes in dendritic cells (DCs) (23) prior to binding to gp130-expressing cells. Formation of the IL-6/IL-6R/gp130 complex initiates intracellular signaling *via* the JAK/STAT pathway, driving phosphorylation of STAT1 and STAT3 proteins (20). The dynamic balance of pSTAT1 and pSTAT3 drives the specificity of the cytokine response and has unique implications for T cell fate and function (24–26). Modulation of this balance occurs with T cell activation and maturation (27) influenced by expression of protein-tyrosine phosphatases (PTPNs) (28), thus illustrating how the combined interplay between components of the IL-6 signaling pathway in specific cell subsets may shape diverging pro-inflammatory properties which may uniquely contribute to IL-6-driven immune dysregulation.

Here, we performed an in-depth characterization of the effect of classical IL-6 signaling in primary human T cells from healthy individuals. We generated a detailed map of IL-6 signaling in CD4^+^ and CD8^+^ naïve and memory T cell subsets, linking mbIL-6R expression levels to the magnitude and balance of phosphorylation of STAT1 and STAT3. In CD4^+^ T cells, increased mbIL-6R was more closely linked to pSTAT1 rather than pSTAT3 responses. Transcriptomic and flow cytometric profiling highlighted a prominent role for IL-6 in early regulation of Tfh characteristics and markers of the apoptotic pathway. Using *in vitro* assays, we further demonstrated the ability of enhanced IL-6 signaling to drive characteristics of multiple T helper cell lineages. Importantly, treatment with tocilizumab in early onset T1D reduced the frequency of CXCR5^hi^PD-1^hi^ Tfh cells confirming an essential role for the IL-6 pathway in human Tfh maintenance and biology. Lastly, rs2228145 genotype data recapitulates enhanced pSTAT1 responses in carriers of the “risk” (mbIL-6R high) allele and highlighted a potential role for the FAS pathway and Tfh1/17 subsets in driving immune dysregulation by enhanced classic IL-6 signaling.

## Materials and Methods

### Human Subjects and Samples

Cryopreserved PBMCs were obtained from adult healthy control participants of the Benaroya Research Institute (BRI) Immune-Mediated Disease Registry selected based on the *IL6R* rs2228145 genotype and no personal or family history of autoimmune disease. When comparing *IL6R* genotypes, samples were age- and sex-matched. For phospho-STAT and total STAT analysis, flow cytometric analysis of marker expression following IL-6 stimulation in the absence of T-cell receptor (TCR) stimulation and comparison of *IL6R* genotypes, samples were homozygous for *PTPN2* rs1893217 (T/T) and *PTPN22* 1858 (C/C). The RNA-seq cohort was not controlled for *PTPN2* rs1893217 (T/T) and *PTPN22* 1858 (C/C). **Supplementary Table 1** lists the individual subjects including demographics, genotypes, and assays performed for each subject. In addition, a *post-hoc* analysis was performed on data generated in the EXTEND Study, a placebo-controlled, double-blinded, randomized clinical trial of adult and pediatric participants with newly diagnosed T1D testing whether tocilizumab would lead to clinical improvements in T1D (29).

### IL-6 Stimulation and Flow Cytometry

**Supplementary Table 2** lists all flow cytometry antibodies described below and includes fluorophore, clone, and source. Frozen human PBMCs were thawed in warm RPMI-1640 (Cytiva HyClone) and 10% FBS (Avantar Seradigm) at 37°C. CD3^+^ T cells were isolated using negative magnetic bead enrichment (Miltenyi Biotec). 5 × 10^5^ CD3^+^ T cells were stimulated with IL-6 (20 ng/mL, BD Biosciences) or left unstimulated and cultured in serum-free media (X-VIVO15, Lonza) for up to 72 h. Cell surface marker staining was performed in PBS for 20 min at room temperature (RT) and cells were then fixed with BD Cytofix for 5 min at RT, stained with PE/Dazzle594-conjugated Streptavidin (BioLegend) if required and stored in FACS buffer (PBS (1X, Cytiva HyClone), bovine serum albumin (BSA, 1%, Sigma-Aldrich), sodium azide NaN3 (0.1%, Sigma-Aldrich) at 4°C until acquisition. Dead cells were excluded using the Zombie-NIR fixable dye (BioLegend). For staining of transcription factors, cells were fixed/permeabilized for 30 min at RT using the Foxp3 Staining Buffer Set (eBioscience/ThermoFisher) and then stained for BCL6, T-bet, RORγt, BATF, IKAROS, and FOXP3 in 1X Perm/Wash buffer (eBioscience/ThermoFisher) for 40 min at RT.

To assess STAT phosphorylation or total STAT levels, 5 × 10^5^ CD3^+^ T cells were resuspended in PBS and stained for viability (Zombie-NIR fixable dye, BioLegend) and selected cell surface markers for 20 min at RT. Cells were washed, resuspended in serum-free media (X-VIVO15, Lonza) and stimulated with IL-6 (20 ng/mL or 1 ng/mL) or left unstimulated for 30 min at 37°C. Cells were fixed with pre-warmed Fix buffer I (BD Biosciences) for 15 min at 37°C and permeabilized using ice-cold Perm buffer III (BD Biosciences) for 30 min on ice. Cells were then stained for remaining markers including CD3, CD4, CD45RA, CD27, CD25, pSTAT3-Y705, STAT1-Y701 or total STAT1 and total STAT3 for 45 min at RT in FACS buffer. All data were acquired on a BD LSRII, BD LSRFortessa or BD FACSCanto II cytometer and analyzed using FlowJo v10.7 (BD Life Sciences).

### T Cell Differentiation

Naïve CD4^+^ T cells were enriched from frozen PBMCs by magnetic bead selection (purity >95%, Miltenyi Biotec). 1 × 10^5^ naïve CD4^+^ T cells were cultured in serum-free media (X-VIVO15, Lonza) in flat-bottom plates pre-coated with anti-CD3 (1 µg/mL, OKT3, BioLegend) in the presence of soluble anti-CD28 (1 µg/mL, CD28.2, BioLegend) and the indicated cytokines for 5 days. For induction of Th17/Tfh characteristics, cells were supplemented with TGF-β1 (0.5 ng/mL, R&D Systems), IL-23 (20 ng/mL, R&D Systems), IL-1β (20 ng/mL, R&D Systems), IL-2 (20 IU/mL), in the presence or absence of IL-6 (20 ng/mL, BD Biosciences). For the Th0 condition, IL-2 was added to the cell culture (20 IU/mL, R&D Systems). After 5 days, the culture supernatants were harvested and analyzed for cytokines using a Luminex assay (R&D Systems). The remaining cells were restimulated with PMA (0.1 µg/mL), and ionomycin (1 µg/mL) in the presence of 1X Brefeldin A (BioLegend) for a total of 4 h at 37°C before surface markers and transcription factors were measured *via* flow cytometry.

### FAS Agonist Treatment

5 × 10^5^ CD3^+^ T cells were cultured in 96-well U-bottom plates in serum-free media (X-VIVO15, Lonza) in the presence or absence of activating anti-human FAS antibody (0.5 µg/mL, CH11, Merck Millipore) for 24 h at 37°C. Cells were stained for viability (Zombie-NIR, BioLegend) and cell surface markers listed in **Supplementary Table 2** in PBS for 20 min, washed with 1X Annexin V Binding Buffer and stained for early apoptotic cells with FITC-conjugated Annexin V in 1X binding buffer (eBioscience/ThermoFisher). Cells were fixed and permeabilized with 1× BD Cytofix/Cytoperm solution for 10 min at RT and subsequently stained for intracellular Caspase 3 in 1× BD Perm/Wash buffer for 20 min at RT.

### RNA Sequencing

CD3^+^ T cells were enriched from frozen PBMCs using negative magnetic bead selection (Miltenyi Biotec) and cultured in media (RPMI-1640, penicillin/streptomycin/glutamine, sodium-pyruvate (all Cytiva HyClone) and 1% human serum (Access Cell Culture)) with or without IL-6 (20 ng/mL, BD Biosciences) for 4 h at 37°C. Cells were then stained for viability in PBS for 10 min with the live/dead blue fixable dye (ThermoFisher) and stained for CD3, CD4, CD8, CD45RA, and CD45RO see **Supplementary Table 2** for 20 min in FACS buffer. Cells were sorted as CD4^+^ naïve (live CD3^+^CD4^+^CD45RA^+^), CD4^+^ memory (live CD3^+^CD4^+^CD45RO^+^) and CD8^+^ naïve (live CD3^+^CD8^+^CD45RA^+^) subsets and 1000 cells of each population were flow sorted and captured in PCR tubes containing 1× reaction buffer (10X lysis buffer, RNase inhibitor, nuclease-free H_2_O, Cell Signaling Technology) and analyzed by RNA-seq as previously described (30). Low-quality libraries (median coefficient of variation (CV) of coverage > 0.9, total reads <2.5 million) were excluded. Counts were then normalized and log_2_ transformed, followed by batch correction using the limma R package (31). Gene set enrichment analysis was performed using the Molecular Signatures Database v7.5.1 (MSigDB) Gene Ontology Biological Processes (GO : BP) collection (32, 33). R software was used to display enriched pathways when indicated.

### Statistics

Statistical testing was performed using GraphPad Prism 8 software. Statistical significance between individual T cell subsets or treatment groups was determined using Mann-Whitney test, Wilcoxon-matched pairs signed rank test or between more than two groups using 2-way ANOVA and Bonferroni’s multiple comparison’s *post-hoc* test. Central horizontal lines in dot and box plots indicate median values.

## Results

### Distinct Patterns of IL-6-Induced STAT1 and STAT3 Phosphorylation in Human T Cell Subsets

**Figure 1:**
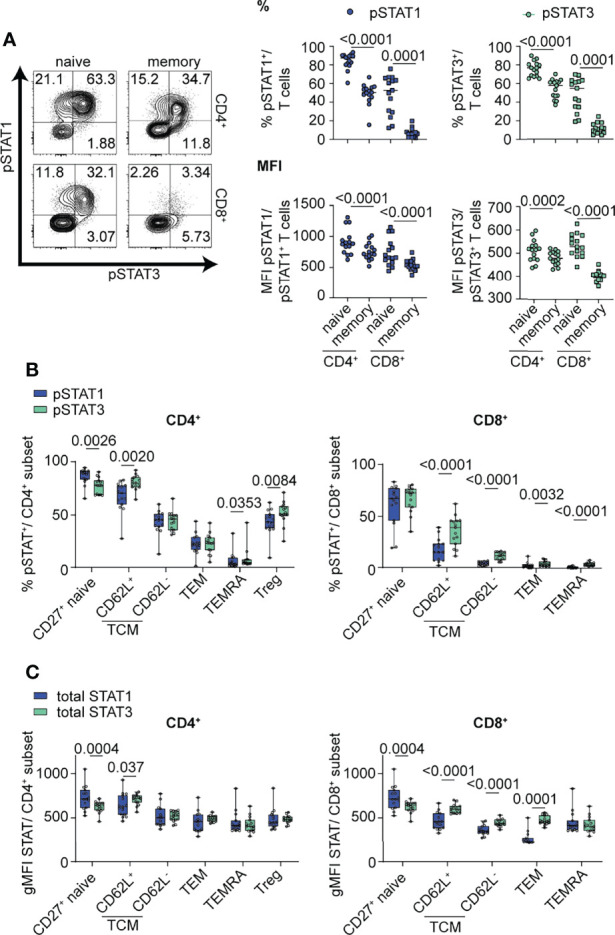
IL-6-driven pSTAT1 and pSTAT3 responses differ in CD4^+^ and CD8^+^ T cell subsets. **(A, B)** CD3-enriched T cells were stimulated with IL-6 (20 ng/mL) for 30 min or **(C)** left unstimulated and the pSTAT1 and pSTAT3 response or levels of total STAT1 and STAT3 protein were determined by flow cytometry. **(A)** Representative flow plot of pSTAT response (left) and frequencies and MFI of gated pSTAT1^+^ or pSTAT3^+^ cells among naïve (CD45RA^+^) or memory (CD45RA^-^) CD4^+^ (non-Treg) and CD8^+^ T cells (right) or **(B)** among CD4^+^ and CD8^+^ T cell subsets in the context of T cell maturation. **(C)** gMFI expression levels of total STAT1 and STAT3 protein were measured in CD4^+^ and CD8^+^ T cell subsets in the context of T cell maturation. (**A–C**) Wilcoxon matched-pairs signed rank test. n = 15.

We first examined the magnitude and balance of STAT1 and STAT3 phosphorylation in response to IL-6 in the context of T cell maturation and lineage. Primary human T cells from healthy control subjects were stimulated with high dose IL-6 (20 ng/ml) for 30 min to ensure robust phosphorylation of both STAT proteins, conditions were based on dose titration (**Supplementary Figure 1A**) and prior time course experiments (24). Relationships between pSTAT responses and sex and age are shown in **Supplementary Table 3**. To minimize the contribution of genetics, we controlled for effect of the Asp358Ala variant in *IL6R* (rs2228145), which we and others have shown to be associated with IL-6R surface expression on T cells; homozygous carriers of the minor C allele have reduced mbIL-6R compared to carriers of the A allele (12, 34). Specifically, all subjects were homozygous for the A allele and did not carry the PTPN2 and PTPN22 risk genotypes, which impact T cell activation and cytokine responses (28, 35). After IL-6 stimulation, there were significant differences for both frequency and median fluorescence intensity (MFI) for both gated pSTAT1^+^ and pSTAT3^+^ cells. Specifically, frequencies of pSTAT1^+^ and pSTAT3^+^ cells were higher in naïve CD4^+^ and CD8^+^ T cell subsets than in the memory subsets (**Figure 1A**). Similarly, pSTAT1 and pSTAT3 MFI were also highest in the naïve subsets (**Figure 1A**). We also detected differences in the patterns of IL-6-induced STAT phosphorylation. Notably, naïve CD4^+^ and CD8^+^ T cells had the highest frequency of pSTAT1 single positive cells whereas memory CD4^+^ T cells had the highest frequency of single positive pSTAT3 cells (**Supplementary Figure 1B**). Naïve CD4^+^ T cells had the highest frequency of pSTAT1 and pSTAT3 double positive cells (**Supplementary Figure 1B**). We also assessed IL-6-induced pSTAT responses in the context of CD4^+^ and CD8^+^ T cell differentiation state including CD27^+^ naïve (CD45RA^+^CD27^+^), T central memory (TCM; CD45RA^-^CD27^+^CD62L^+^ and CD62L^-^), T effector memory (TEM; CD45RA^-^CD62L^-^CD27^-^), terminally differentiated effector memory T cells (TEMRA; CD45RA^+^CD27^-^) and CD4^+^ Tregs (CD4^+^CD25^hi^) (**Figure 1B** and **Supplementary Figure 1C**). Individual subsets showed striking differences in the magnitude of their response to IL-6, as measured by frequency of pSTAT^+^ cells, which decreased from naïve to terminally-differentiated memory T cell subsets (**Figure 1B**). Although all subsets phosphorylated STAT1 and STAT3 in response to IL-6, there was heterogeneity in the balance of phosphorylation with STAT1 phosphorylation predominant in the naïve CD4^+^ T cell subset and STAT3 phosphorylation predominant in CD4^+^CD62L^+^ TCM, CD4^+^ Treg and memory CD8^+^ T cell subsets (**Figure 1B**).

To determine if total STAT protein levels contribute to the observed differences in pSTAT levels in specific T cell subsets, we measured the gMFI of total STAT1 and STAT3 in unstimulated cells at steady state *via* flow cytometry (**Figure 1C**). Individual subsets showed heterogeneous levels of total STAT proteins, including higher expression of total STAT1 protein in naïve CD4^+^ and CD8^+^ subsets, while total STAT3 had higher expression in CD8^+^ memory subsets (**Figure 1C**). Although our data shows that availability of total STAT protein is different between T cell subsets and may drive competitive phosphorylation of one STAT over the other, our data suggests that additional components to total STAT proteins influence the magnitude and balance of STAT1 and STAT3 phosphorylation (**Figure 1C**).

**Figure 2:**
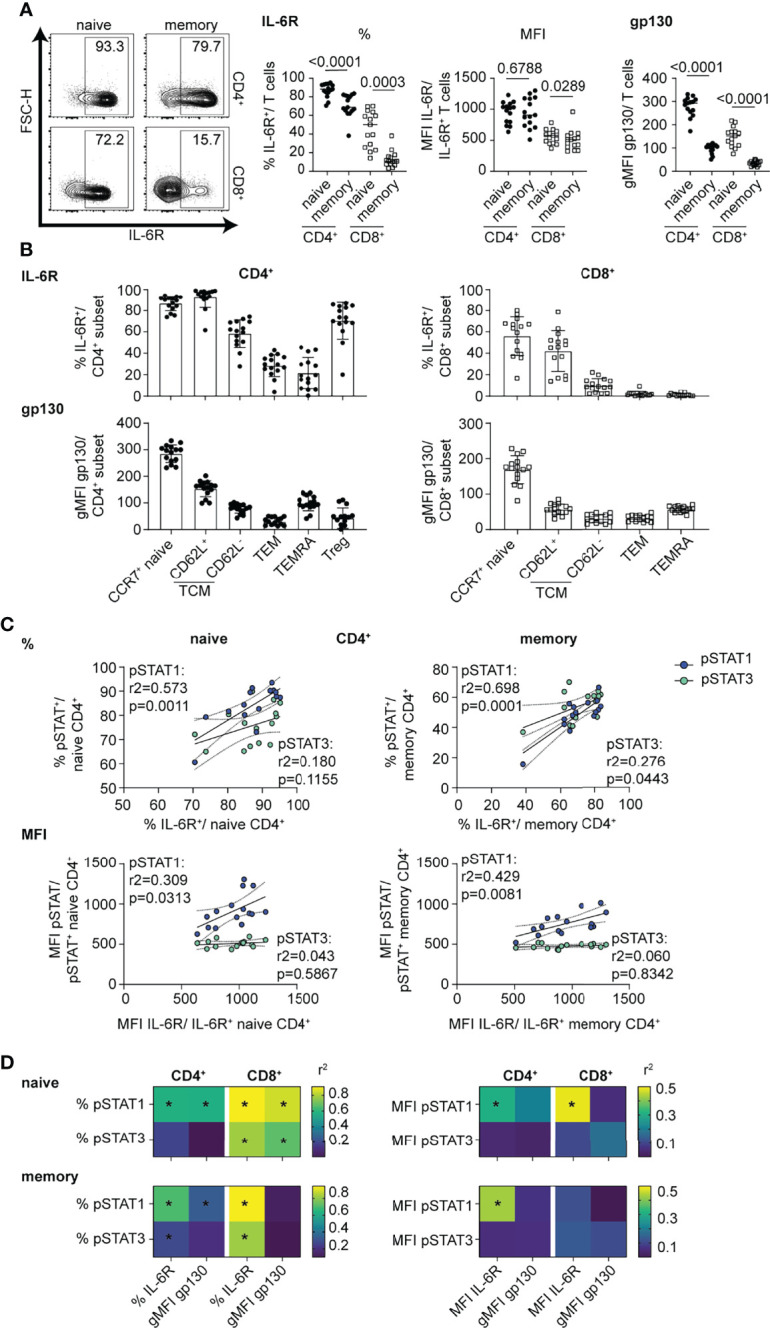
mbIL-6R levels direct strength of pSTAT1 signaling response in CD4^+^ and CD8^+^ T cell subsets. **(A-D)** CD3-enriched T cells were left unstimulated or **(C, D)** stimulated with IL-6 (20 ng/mL) for 30 min or and mbIL-6R and gp130 levels or the pSTAT1 and pSTAT3 response were determined by flow cytometry. **(A)** Representative flow plot of mbIL-6R expression and frequencies and MFI of gated mbIL-6R^+^ cells (middle) or gMFI expression levels of gp130 (right) among naïve or memory CD4^+^ (non-Treg) and CD8^+^ T cells or **(B)** CD4^+^ and CD8^+^ T cell subsets in the context of T cell maturation. **(C)** Simple linear regression analysis of expression levels as frequencies (%, top) or MFI (bottom) of gated mbIL-6R^+^ and pSTAT1^+^ or pSTAT3^+^ cells in naïve or memory CD4^+^ (non-Treg) T cells. **(D)** Summary data of simple linear regression analysis of expression levels as frequencies (left) or MFI (right) of gated mbIL-6R^+^ or the gMFI of gp130 and pSTAT1^+^ or pSTAT3^+^ cells in naïve or memory CD4^+^ (non-Treg) and CD8^+^ T cells. **(A)** Wilcoxon matched pairs signed rank test. **(C, D)** Linear regression analysis with Pearson’s correlation coefficients. n = 15.

We next investigated whether these subset-specific differences in the magnitude and balance of STAT1 and STAT3 phosphorylation were due to different levels of components of the IL-6R complex. We found that the frequency of mbIL-6R^+^ cells and gp130 expression levels were significantly higher in naïve versus memory subsets, yet among gated mbIL-6R^+^ cells there was no difference in mbIL-6R MFI in these subsets (**Figure 2A**). There were more mbIL-6R^+^ cells in the CD4^+^ T cell subsets compared to the CD8^+^ T cell subsets (**Figure 2A**). Across the CD4^+^ T cell subsets, CCR7^+^ naïve T cells, CD62L^+^ TCM, and Treg cells had the highest frequency of mbIL-6R^+^ cells while gp130 expression levels were highest in CCR7^+^ naïve cells but relatively low in the other memory subsets and Treg cells (**Figure 2B**). Furthermore, linear regression analyses revealed that the frequency of mbIL-6R^+^ cells and the frequency of pSTAT1^+^ and pSTAT3^+^ cells was positively correlated in memory CD4^+^ T cells (**Figures 2C, D** **)**, naïve CD8^+^ T cells (**Supplementary Figure 1D**) and memory CD8^+^ T cells (**Supplementary Figure 1D**), consistent with previous studies (34). There was no significant correlation between the frequency of mbIL-6R^+^ cells and the frequency of pSTAT3^+^ cells in the naïve CD4^+^ T cell compartment (**Figure 2C**). Notably in naïve CD4^+^ T cells, mbIL-6R MFI correlated with pSTAT1 MFI but not with pSTAT3 MFI (**Figure 2C**). The lack of correlation between mbIL-6R MFI and pSTAT3 MFI extended to CD4^+^ memory and CD8^+^ naïve T cells (**Figure 2C** and **Supplementary Figure 1D**), suggesting that pSTAT1 levels in response to IL-6 are more sensitive to mbIL-6R expression than pSTAT3 levels. Interestingly, linear regression analysis of the frequency but not MFI of pSTAT1^+^ cells with gp130 expression levels revealed similar relationships as observed with IL-6R expression and pSTAT responses in naïve CD4^+^, memory CD4^+^ and naïve CD8^+^ T cells (**Figure 2D**).Together these findings demonstrate that the magnitude and balance of STAT1 and STAT3 phosphorylation following IL-6 stimulation is specific to T cell subset and associated with cell surface expression of components of the IL-6R, mbIL-6R and gp130.

### T Cell Maturation and Lineage Influence the Transcriptomic Response to IL-6

**Figure 3:**
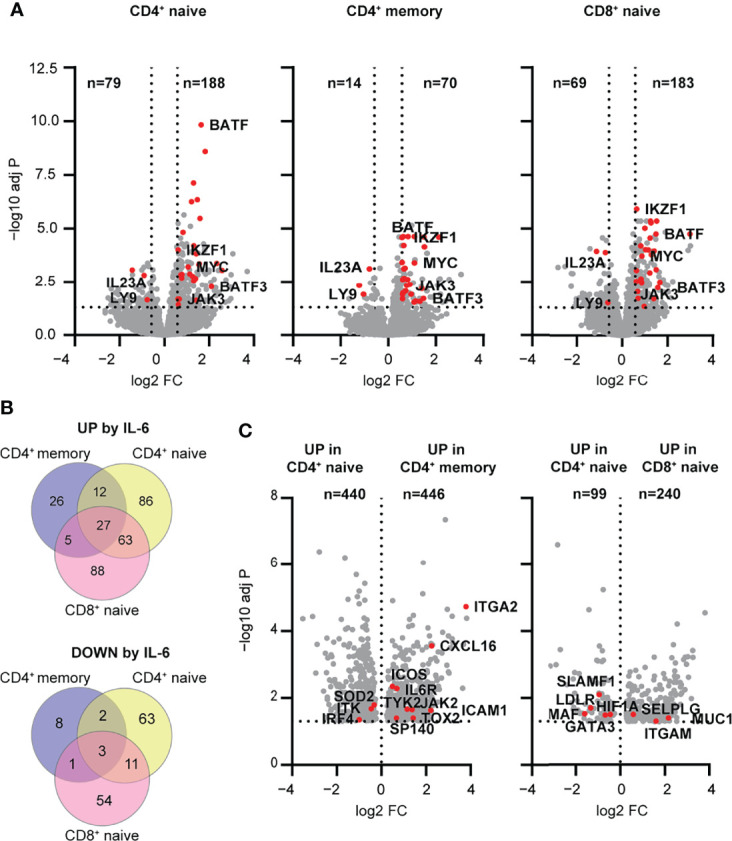
Early transcriptomic response to IL-6 stimulation highlights pathways regulated by mbIL-6R signaling in human T cell subsets. **(A-C)** Transcriptome profiling of total CD4^+^ naïve, memory, and CD8^+^ naïve T cell subsets stimulated with IL-6 (20 ng/mL) or left unstimulated for 4 h was performed using RNA-seq. **(A)** Volcano plots showing differentially expressed genes between IL-6 stimulated versus unstimulated T cells. Shared up- and downregulated genes between subsets are highlighted (red). **(B)** Venn diagram of IL-6 up- (top) or downregulated (bottom) genes unique to or shared between T cell subsets. **(C)** Genes upregulated following IL-6 stimulation but not at baseline comparing CD4^+^ naïve versus memory or CD4^+^ naïve versus CD8^+^ naïve T cells. **(A, B)** FC > 1.5, FDR adj. P < 0.05. **(C)** FDR adj. P < 0.05. n = 16.

We next examined the transcriptomic response to IL-6 signaling to address how the differences in the magnitude and balance of STAT1 and STAT3 phosphorylation between T cell subsets were reflected in the transcriptome signature. We performed RNA-seq on sorted CD4^+^ naïve and memory T cells, as well as CD8^+^ naïve T cells, at both baseline and after a 4 h stimulation with IL-6 (20 ng/ml). CD8^+^ memory T cells were not included given their weak response to IL-6. We found that the total number of differentially expressed genes in response to IL-6 were comparable between the naïve CD4^+^ and CD8^+^ T cells with 267 and 252 genes, respectively (**Figure 3A**). However, the number of differentially expressed genes in memory CD4^+^ T cells was much lower with a total of 84 genes (**Figure 3A**). This difference between naïve and memory subsets is consistent with the magnitude of pSTAT responses to IL-6 in naïve versus memory subsets shown in **Figure 1A**. Overall, a set of 30 genes were conserved among all three T cell subsets with 27 up-regulated genes and 3 down-regulated genes, including known IL-6 target genes, *MYC* and *JAK3* (**Figure 3B** and **Supplementary Tables 4, 5**). Gene ontology (GO) analysis of shared up- and downregulated genes highlighted a role for cell differentiation processes mainly driven by transcription factors associated with T cell differentiation including *BATF*, *BATF3*, and *IKZF1* (IKAROS) (**Supplementary Figure 2A**). Interestingly, two genes involved in the Th17 differentiation pathway (*IL23A*, *LY9*) were downregulated by IL-6 stimulation, suggesting a potential ambiguous role for IL-6 signaling in this pathway. Other transcription factors involved in Tfh (i.e., *BCL6*) or Th17 (i.e., *HIF1A*) differentiation showed upregulation in all T cell subsets; however, this did not meet significance or fold change -thresholds in all examined subsets. This was also observed for other IL-6 target genes (*SELL*, *CCR7*, *STAT3*, *SOCS3, LDLR*, and *LAG3*).

To better understand the relative differences in IL-6-dependent gene regulation in the context of maturation state, specifically CD4^+^ naïve T cells versus CD4^+^ memory T cells, and in the context of lineage, specifically CD4^+^ naïve T cells versus CD8^+^ naïve T cells, we analyzed the genes that were differentially upregulated in these subsets following stimulation. In the context of maturation state, CD4^+^ naïve T cells upregulated genes related to metabolic processes and ribosome biogenesis, whereas CD4^+^ memory T cells showed relative enrichment of genes related to immune function including *ICOS*, *TOX2*, *SP140* and *CXCL16* (**Figure 3C** and **Supplementary Tables 6, 7** **)**. Comparison of CD4 and CD8 lineages showed that CD4^+^ naïve T cells were enriched for upregulated genes related to cell differentiation and signaling (*MAF*, *HIF1A*, *GATA3*), while CD8^+^ naïve T cells were enriched in upregulated genes related to cell localization and adhesion (*ITGAM*, *SELPLG*) (**Figure 3C** and **Supplementary Tables 8, 9**). Collectively, these findings suggest that the magnitude and quality of response to IL-6 signaling is dependent on maturation state and lineage. In addition, they support a role for IL-6 signaling in driving features of T cell differentiation (e.g., Th17 and Tfh) pathways that have been implicated in early immune dysregulation, even in the absence of TCR stimulation.

### mbIL-6R Signaling Drives T Cell Phenotypes Linked to Autoimmunity

**Figure 4:**
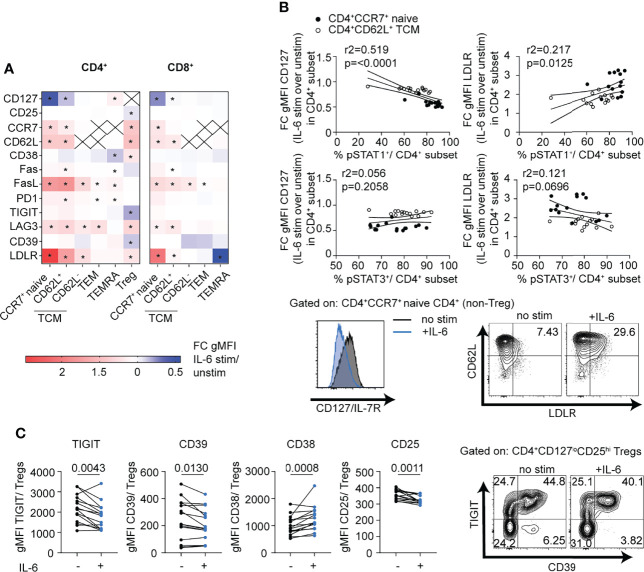
mbIL-6R signaling induced pSTAT1 responses are linked to T cell subset specific phenotypic changes. CD3-enriched T cells were stimulated with IL-6 (20 ng/mL) or left unstimulated for 24 h, and then change in expression levels of selected surface markers was determined *via* flow cytometry. **(A)** Heatmap showing the fold change in surface gMFI expression levels of selected markers following IL-6 stimulation over the unstimulated control in distinct CD4^+^ and CD8^+^ T cell subsets. **(B)** Simple linear regression analysis of the fold change in CD127 (left) and LDLR (right) expression and the magnitude of the pSTAT1 (top) and pSTAT3 (bottom) responses following IL-6 stimulation in naïve CD4^+^ (black dots) or CD62L^+^ TCM (white dots) cells and representative flow plot of IL-6 driven changes in CD127 and LDLR expression on gated naïve CD4^+^ T cells (bottom). **(C)** Paired analysis of gMFI expression levels of TIGIT, CD39, CD38, and CD25 among gated CD127^lo^CD25^hi^ Treg cells in the presence (+) or absence (-) of IL-6 stimulation. **(A)** 2-way ANOVA and Bonferroni’s multiple comparisons test, * FDR p < 0.05. **(B)** Linear regression analysis with Pearson’s correlation coefficients. **(C)** Wilcoxon matched-pairs signed rank test. n = 14-15.

While the RNA-seq data provided a broad snapshot of the early transcriptomic response to IL-6 signaling, we were interested to see if this translated to changes in protein expression. CD3-enriched T cells were incubated for 24 h in the presence or absence of IL-6 (20 ng/ml), and the response to IL-6 was analyzed in both the CD4^+^ and CD8^+^ compartments in gated naïve (CD45RA^+^CCR7^+^ or CD27^+^), TCM (CD45RA^-^CCR7^+^ or CD27^+^), TEM (CD45RA^-^CCR7^-^ or CD27^-^), TEMRA (CD45RA^+^CCR7^-^ or CD27^-^) and Treg (CD127^low^CD25^hi^) to match studies shown in **Figure 1**. Note, CCR7 and CD27 were used in different panels as equal markers to define these subsets. Consistent with the RNA-seq data, CD62L, CCR7 and LDLR cell surface levels were significantly increased in response to IL-6 in both CD4^+^ and CD8^+^ naïve and TCM subsets as well as the CD4^+^ Treg compartment (**Figure 4A**). In these same T cell subsets, there was also upregulation of the apoptotic marker FASL and the inhibitory receptor LAG-3 and downregulation of CD127 while FAS was strongly upregulated in CD4^+^CD62L^+^ TCM cells (**Figure 4A** and **Supplementary Figure 2B**). Notably, the strength of change for these markers was dependent on T cell differentiation stage and paralleled observed differences in the pSTAT signaling strength with strongest response in the naïve subsets and a weaker response in the memory subsets (**Figure 4A**). This was most pronounced for LDLR and CD127, and linear regression analysis established a direct link with the magnitude of the pSTAT1 but not pSTAT3 response (**Figure 4B**). CD4^+^ Tregs also upregulated CCR7, CD62L, LAG-3 and LDLR in response to IL-6 similar to the naïve and memory subsets, with additional unique protein expression changes including upregulation of CD38 and downregulation of CD25, TIGIT, and CD39 (**Figure 4A**, **Supplementary Figure 2B**). Given the importance of CD39, TIGIT, and CD25 as markers of highly suppressive Treg cells (36, 37), we did a paired analysis of the geometric mean fluorescence intensity (gMFI) in gated CD127^low^CD25^hi^ Treg cells at baseline and in the presence of IL-6 (**Figure 4C**). Consistent with our unsupervised analysis, IL-6 induced upregulation of CD38 and downregulation of CD25, CD39, and TIGIT (**Figure 4C**).

**Figure 5:**
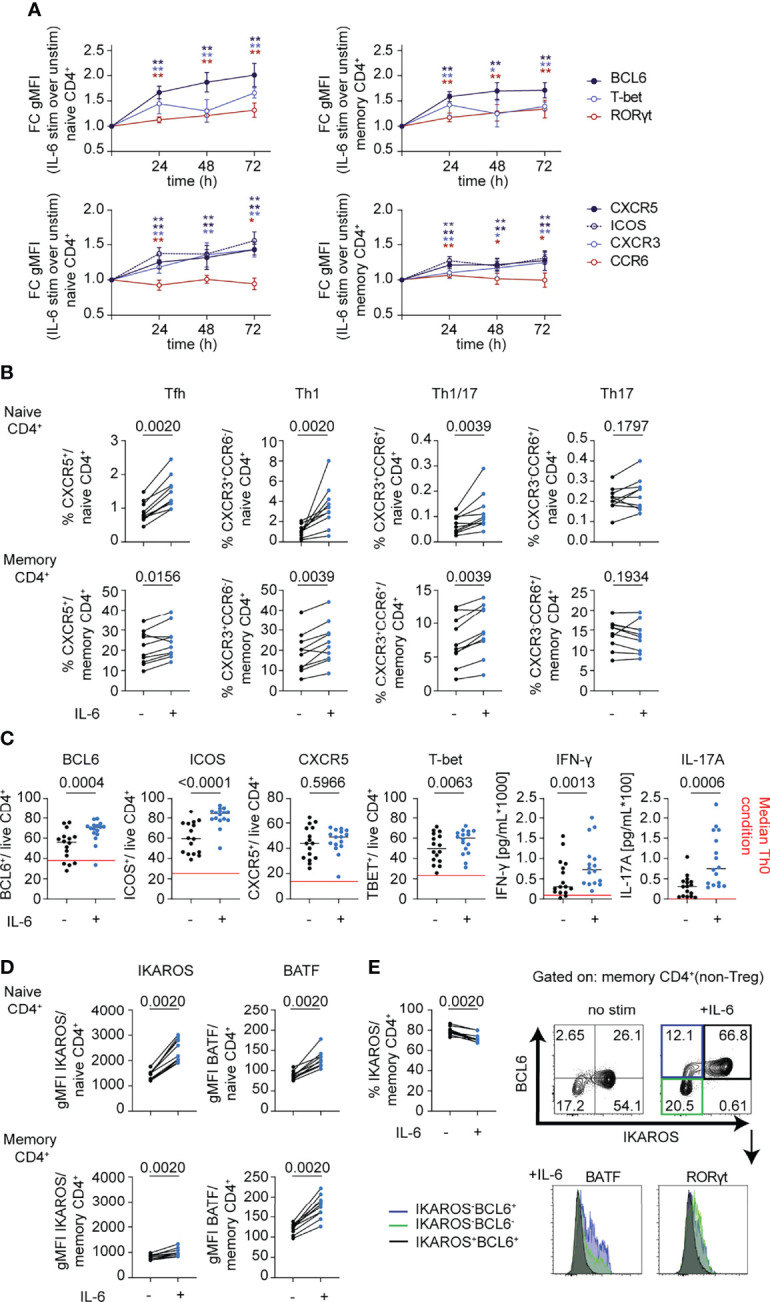
mbIL-6R signaling drives phenotypic changes consistent with features observed in autoimmunity. **(A, B, D)** CD3-enriched T cells were stimulated with IL-6 (20 ng/mL) or left unstimulated for up to 72 h and the change in expression levels of selected surface and intracellular markers was determined *via* flow cytometry. **(A)** Fold change in gMFI expression levels of BCL6, RORγt, and T-bet (top) or of Th-related surface receptors (bottom) in naïve (CD45RA^+^) or memory (CD45RA^-^) CD4^+^ (non-Treg) T cells following IL-6 stimulation compared to unstimulated controls over time. **(B)** Paired analysis of the frequency of gated Th subsets in total naïve (CD45RA^+^) or memory (CD45RA^-^) CD4^+^ (non-Treg) T cells in IL-6 stimulated (+) or unstimulated (-) cells after 72 h of culture. **(C)** Naïve (CD45RA^+^) CD4^+^ T cells were cultured in conditions used to differentiate both Tfh and Th17 cells (IL-1β, IL-2, IL-23, TGF-β, α-CD3/CD28) in the presence (+) or absence (-) of IL-6 (20 ng/mL) for 5 days and changes in surface (CXCR5, ICOS) or intracellular markers (BCL6, T-bet) were determined *via* flow cytometry on gated live CD4^+^ T cells. Changes in cytokines from culture supernatants were detected by Luminex assay. Red line indicates median levels measured in Th0 conditions. **(D)** gMFI expression levels of BATF and IKAROS in gated naïve (CD45RA^+^) or memory (CD45RA^-^) CD4^+^ (non-Treg) T cells in IL-6 stimulated (+) or unstimulated (-) cells after 72 h of culture. **(E)** Frequency of IKAROS^+^ in memory CD4^+^ T cells as in **(D)** and representative flow plot of BCL6 and IKAROS expression in memory CD4^+^ T cells (top) and histogram of expression levels of BATF and RORγt among gated subsets defined by IKAROS and BCL6 expression. **(A)** Wilcoxon matched-pairs signed rank test comparing IL-6 stimulation to unstimulated control condition for each sample and each time-point. *p < 0.05, **p < 0.01 **(B–E)** Wilcoxon matched-pairs signed rank test. **(A, B, D, E)** n = 10. **(C)**. n = 16.

In view of the enrichment of transcription factors involved in T-helper cell differentiation pathways in the RNA-seq dataset and implications for IL-6-driven pathogenic Th1 (38), Th17 (39), and Tfh (40) responses in autoimmunity, we focused on CD4^+^ T cells to better understand the impact of classic IL-6 signaling on T-helper cell subsets in the absence of TCR stimulation. We followed expression of T-helper-related transcription factors, BCL6, BATF, RORγt, T-bet, and IKAROS, and surface receptors, CXCR5, ICOS, CXCR3, and CCR6, after IL-6 stimulation over a 72-h period in CD4^+^ naïve and memory cells. Here, we observed increased expression of all the transcription factors examined with BCL6, the Tfh regulator having the greatest increase in both subsets (**Figure 5A**). Consistent with this observation, surface expression of the Tfh cell markers CXCR5 and ICOS was increased in response to IL-6 (**Figure 5A**). Pronounced increases were also observed for the Th1 markers T-bet and its target gene, CXCR3 (**Figure 5A**). IL-6 also affected expression of Th17 genes, but to a lesser extent with only a modest increase in the levels of the transcription factor RORγt and a subtle but significant decrease on cell surface expression of CCR6 (**Figure 5A**). Overall, gMFI changes of these markers translated into increased frequencies of gated Tfh, Th1, and Th1/17 subsets among both naïve and memory CD4^+^ T cells (**Figure 5B** and **Supplementary Figure 2C**). While IL-6 signaling has been shown to be critical for stable TCR-dependent differentiation of murine naïve CD4^+^ T cells into Th17 (5) and germinal center (GC)-Tfh cells (6, 7), many studies have highlighted redundant roles of different cytokines and STAT proteins in human Th17/Tfh differentiation pathways (10, 11, 41). Using naïve CD4+T cells in differentiation assays under conditions driving features of both Tfh and Th17 cells, we demonstrated that the presence of IL-6 enhances features of Tfh, Th1, and Th17 cells by increasing the frequency of BCL6, ICOS, T-bet, IFN-γ, and IL-17A positive cells (**Figure 5C**).

In line with the RNA-seq dataset, we detected significant increases in protein expression levels of BATF and IKAROS (**Figure 5D**). Yet, the frequency of IKAROS^+^ cells significantly decreased among CD4^+^ memory cells, suggesting a selective loss among a small subset of cells following IL-6 stimulation (**Figure 5E**). Importantly, IKAROS expression was restricted to BCL6^+^ cells following IL-6 stimulation in memory subsets but showed reciprocal expression with BATF and RORγt, which were uniquely expressed among subsets of either IKAROS^-^BCL6^+^ cells or IKAROS-BCL6^-^ cells (**Figure 5E**). Collectively, these data corroborate the pleiotropic character of IL-6 by enhancing features of multiple Th cell lineages, most prominently of Tfh cells, and likely amplifying and integrating additional pathways on top of dominant lineage-defining cytokines. Furthermore, these data suggest that mbIL-6R signaling induces highly heterogeneous responses depending on lineage and differentiation stage and may reflect differences in the IL-6 signaling strength and pSTAT response.

### Tocilizumab Treatment Impairs Tfh Compartment and FAS Expression

**Figure 6:**
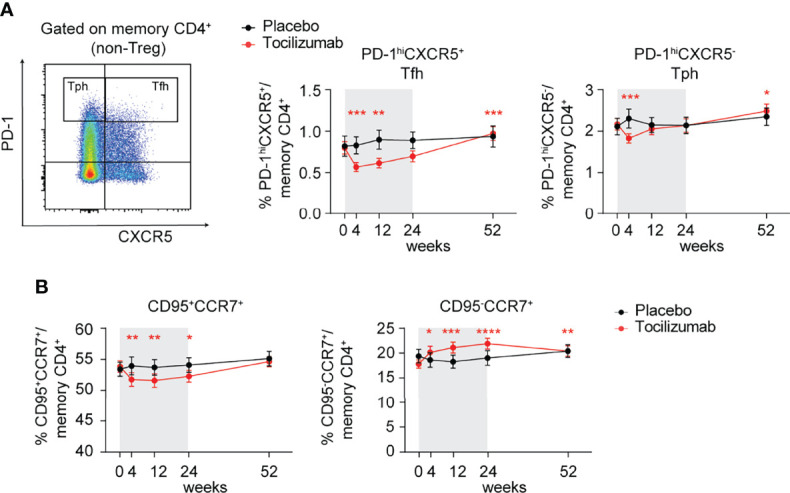
Tocilizumab treatment decreases the frequency of PD-1^hi^CXCR5^+^ Tfh cells and modulates FAS/CD95 expression on memory CD4^+^ T cells. **(A, B)** Individuals enrolled in the EXTEND study were treated with tocilizumab (red line) or assigned to the placebo group (black line) and changes in T cell phenotypes were assessed over time *via* flow cytometry. **(A)** Representative flow plot (left) of gating strategy to define PD-1^hi^CXCR5^+^ Tfh and PD-1^hi^ CXCR5^-^ Tph cells and (right) changes in frequencies of Tfh and Tph cells over time. Time frame of tocilizumab treatment during 24 weeks is indicated by gray shading. **(B)** Frequencies of CCR7^+^CD95^+^ and CCR7^+^CD95^-^ memory CD4^+^ (non-Treg) T cells over time in the tocilizumab-treated versus placebo group. **(A, B)** Plots show mean values and error bars indicate the standard error of the mean (SEM). Significance between pre-treatment baseline (week 0) and respective visits was calculated through the Wilcoxon matched pairs signed rank test. **(A)** n = 23-26 (placebo group); n = 50-53 (tocilizumab group). **(B)** n = 24-26 (placebo group); n = 50-53 (tocilizumab group).

To determine the impact of mbIL-6R blockade on IL-6-related T cell phenotypes *in vivo*, we analyzed data from the EXTEND clinical trial, which investigated the impact of tocilizumab, a monoclonal antibody targeting global IL-6 signaling by blocking both mbIL-6R and soluble IL-6R, on beta cell loss in pediatric patients with new onset T1D (29). The EXTEND trial reported a lack of clinical efficacy and no significant changes in the frequency of FOXP3^+^ Treg, IL-17^+^ Th17, and IL-21^+^ Tfh cells. In our *post-hoc* analysis, we did not find differences in Tfh based on CXCR5 gating alone (**Supplementary Figure 2D**) but detected a significant decrease in the frequency of CXCR5^+^PD^-^1^hi^ Tfh cells among CD4^+^ memory T cells in the treatment but not in placebo group at weeks 4 and 12 during the 24-week treatment course (**Figure 6A**). In contrast, CXCR5^-^PD^-^1^hi^ T peripheral helper cells (Tph), a subset associated with progression to T1D (42) in autoantibody-positive children, only showed a transient significant reduction at week 4 post-treatment, which normalized to levels detected in the placebo group at later time points (**Figure 6A**). Interestingly, both subsets significantly increased compared to baseline in the treatment but not placebo group 28 weeks following the last treatment with tocilizumab (**Figure 6A**).

Given our findings implicating IL-6 signaling in regulating FAS, we assessed FAS expression levels among CD4^+^ memory T cells over time. Compared to placebo, tocilizumab treatment resulted in a significant reduction in the frequency of CD95^+^CCR7^+^ and conversely an increase in the frequency of CD95^-^CCR7^+^ cells in the CD4^+^ memory T cell compartment (**Figure 6B**), supporting a role for IL-6-driven maintenance of FAS expression on CD4^+^ memory T cells. Together, these results suggest an important role for T cell intrinsic IL-6 signaling in the maintenance of CXCR5^+^PD-1^hi^ Tfh cells and FAS expression *in vivo*, but that these alterations alone are not adequate to alter the progression of new onset T1D.

### mbIL-6R Genotype Influences IL-6 Response and FAS-Induced AICD

**Figure 7:**
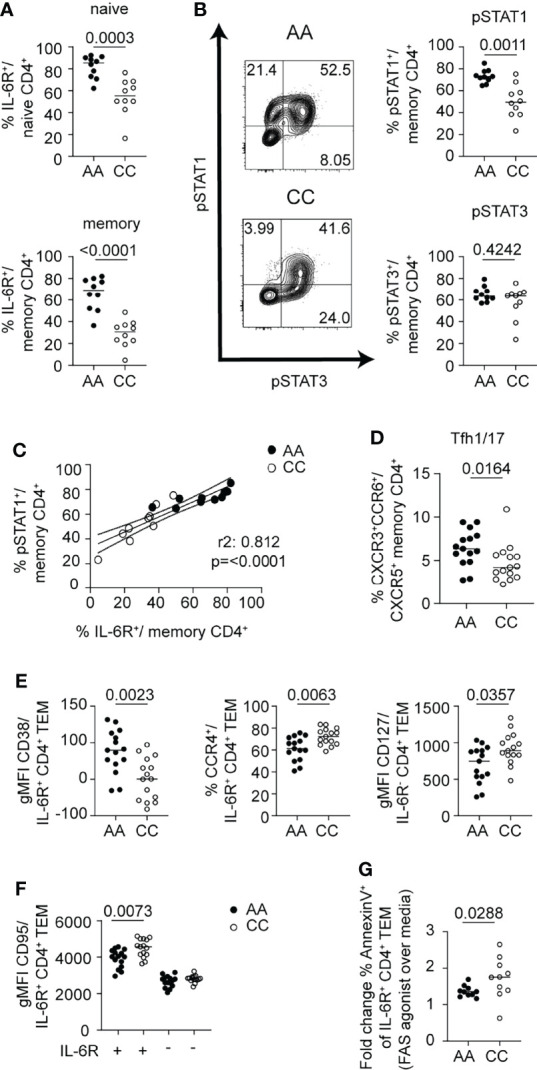
mbIL-6R rs2228145 genotype determines pSTAT1 response to IL-6 and is linked to *ex vivo* phenotypic differences in CD4^+^ T cells. CD3-enriched T cells from carriers of the mbIL-6R^hi^/A or mbIL-6R^lo^/C allele were **(A, C–F)** left unstimulated or **(B, C)** stimulated with IL-6 (20 ng/mL) for 30 min and surface markers or the pSTAT1 and pSTAT3 response were determined by flow cytometry. **(A)** Frequency of gated mbIL-6R^+^ cells at baseline in naïve (CD45RA^+^) or memory (CD45RA^-^) CD4^+^ (non-Treg) T cells. **(B)** Representative flow plots (left) and summary data (right) of the frequencies of pSTAT1^+^ (top) and pSTAT3^+^ (bottom) cells in memory (CD45RA^-^) CD4^+^ (non-Treg) T cells. **(C)** Simple linear regression analysis of the frequencies of mbIL-6R^+^ and pSTAT1^+^ memory CD4^+^ (non-Treg) T cells. **(D)** Frequency of Tfh1/17 cells among memory CD4^+^ (non-Treg) T cells or **(E, F)** gMFI expression levels of CD38, CCR4, CD127 and CD95/FAS in gated mbIL-6R^+^ or mbIL-6R^-^ TEM cells after 24 h in culture in the absence of stimulation. **(G)** CD3-enriched T cells were treated with agonistic FAS antibody or left untreated for 24 h and the change in the frequency of Annexin V^+^ cells in gated CD4^+^CD45RA^-^CD27^-^CD62L^-^IL-6R^+^ TEM cells was determined by flow cytometry. **(A, B, D–G)** Mann-Whitney test. **(C)** Linear regression analysis with Pearson’s correlation coefficients. **(A–C, G)** Pooled data from 2 independent experiments. Data from one experiment. **(A)** n = 20, (**B, C)** n = 9-10, (**D–F)** n = 15, **(G)** n = 10.

To extend our findings in the context of differences in mbIL-6R expression based on genetic variants, we compared healthy control subjects homozygous for the *IL6R* variant (rs2228145) C allele and healthy carriers of the A allele; subjects were age- and sex-matched. We first confirmed significantly lower surface IL-6R levels on CD4^+^ naïve and memory T cell subsets from subjects with the CC genotype (**Figure 7A**). There was a striking difference in response to IL-6 between these two genotypes with the AA genotype having significantly more pSTAT1^+^ cells in both the CD4^+^ naïve and memory compartments than the CC genotype, after 30 min of IL-6 stimulation (**Figure 7B** and **Supplementary Figure 2E**). In contrast, there was no difference in the frequency of pSTAT3^+^ cells between the AA and CC genotypes (**Figure 7B** and **Supplementary Figure 2E**). Furthermore, the frequency of mbIL-6R^+^ cells positively correlated with the frequency of pSTAT1^+^ cells in the CD4^+^ memory compartment (**Figure 7C**), further underscoring the link between mbIL-6R levels and STAT1 phosphorylation.

We next asked whether these early differences in mbIL-6R expression and the magnitude of pSTAT signaling responses translated into phenotypic differences in memory T cells directly *ex vivo*, focusing on cell surface markers that we had shown changed in response to IL-6 *in vitro*. Here, we found that the percentage of Tfh1/17 cells among total memory CD4^+^ T cells was significantly higher in the AA genotype compared to the CC genotype (**Figure 7D**). Within the CD4^+^ TEM compartment of the AA genotype carriers, the expression of CD38 was increased and CCR4 decreased on mbIL-6R^+^ cells compared to carriers of the CC genotype. In addition, carriers of the AA genotype had lower CD127/IL7R expression in IL6R^-^ TEM cells than the CC subjects (**Figure 7E**). Thus, T cells of individuals with the AA genotype show a greater degree of IL-6-driven phenotypic characteristics in comparison to those of individuals with the CC genotype. Collectively, these genotype data corroborate a link for enhanced pSTAT1 responses with high mbIL-6R expression levels.

There was a marked effect of the *IL6R* genotype on FAS expression. Our flow analysis of CD4^+^ T cells after exposure to IL-6 showed an increase in FAS. Yet, we detected significantly lower levels of FAS expression on IL-6R^+^ CD4^+^ TEM cells in AA carriers compared to CC carriers; in contrast, FAS gMFI was not different between genotypes in IL-6R^-^ CD4^+^ TEM cells (**Figure 7F**). Given the prominent role of FAS in promoting apoptosis through activation-induced cell death (AICD) *via* its ligand FASL, we investigated whether IL6R^+^ TEM cells from CC subjects were more susceptible to AICD. We incubated CD3-enriched T cells from healthy control subjects selected for high (CC genotype) or low (AA genotype) FAS expression among IL6R^+^ CD4^+^ TEM cells with an agonistic FAS antibody (43) and measured markers of apoptosis after 24 h of incubation (**Figure 7G**). Indeed, we found a higher fold change of Annexin V^+^ cells comparing the FAS agonist over the media alone condition in the CC genotype (**Figure 7G**). These findings provide a potential mechanistic link on how this SNP confers protection for autoimmune diseases by potentially depleting autoreactive pathogenic T cells *via* AICD. Subtle differences in the phenotype of the CD4^+^ T cells, specifically TEM cells, highlight IL-6-regulated pathways of interest, including the apoptotic pathway that may contribute to observed association of this *IL6R* SNP for development of autoimmunity.

## Discussion

In this study, we find that expression levels of components of the IL-6R complex differed between both T cell lineages and maturation stages. We found a maturation stage-dependent decline in mbIL-6R-expressing cells that mirrored a parallel decline and an alteration in the balance of STAT responses in more differentiated CD4^+^ and CD8^+^ subsets. This confirms and extends studies showing that T cell activation alters mbIL-6R responsiveness through ADAM-17-mediated shedding and reduces sensitivity to classic IL-6 signaling (44, 45).

Our data demonstrate that the magnitude of pSTAT1 responses is tightly linked to mbIL-6R levels, which are higher in CD4^+^ T cells compared to CD8^+^ T cells and in naïve compared to terminally differentiated memory subsets. Following IL-6 stimulation, we found a stronger induction of pSTAT1 over pSTAT3, specifically in naïve CD4^+^ T cells, which was closely linked to mbIL-6R levels, suggesting a dominant role for an IL-6-induced pSTAT1 response in naïve CD4^+^ T cells. This is consistent with a previous study demonstrating that the induction of PTPNs through T cell activation influences the level of pSTAT1 in response to IL-6 in memory CD4^+^ T cells (28). Our data further demonstrates that other components of the IL-6 signaling pathway, including the availability of total STAT1 and STAT3 as well as gp130 may influence the pSTAT output in different T cell subsets beyond mbIL-6R expression. This may extend to additional factors including expression of JAK proteins, SOCS proteins and other PTPNs. The relationship of pSTAT1 with increased mbIL-6R is further supported by: (1) our flow cytometry data, which demonstrated a link between pSTAT1 levels and expression of the IL7R and LDLR; and (2) our genotype data with carriers of the “risk” IL-6R coding variant rs2228145 (AA) showing a stronger pSTAT1 response compared to carriers of the non-risk allele (CC).

Moreover, these data confirm observations from mouse T cells showing a dominant role of pSTAT1 specifically in naïve CD4^+^ T cells following IL-6 stimulation (28). Our finding that the balance of phosphorylation of STAT1 and STAT3 is dependent on human T cell subsets is of particular interest given recent advances in defining the role of each STAT protein in driving the cytokine signature, where STAT3 drives the overall transcriptomic output while STAT1 diversifies the response (24). Interestingly, STAT1 binding to chromatin was dramatically reduced in the absence of STAT3, and the remaining STAT1-bound regions were largely found in the proximity of genes downregulated by IL-6 stimulation (24). Our dataset supports a similar role for STAT1 in broadening the response to IL-6 as well as negatively regulating the expression of a set of genes in human naïve CD4^+^ T cells. Hence, our findings suggest that IL-6-induced STAT1 may promote early events that govern the transition into pathogenic T cells driving autoimmunity.

Our findings highlight several key aspects of the IL-6 pathway on the promotion of human T cell phenotypes with implications for autoimmune diseases. First, our results suggest that the IL-6 pathway plays a dominant role in priming and maintaining a Tfh program. The mbIL-6R signaling pathway, even in the absence of TCR stimulation, promotes features of Tfh cells, in both naïve and memory CD4^+^ T cells, including the induction of BCL6, CXCR5, and ICOS. Additionally, IL-6 stimulation decreased IL-7R expression, a pathway shown to repress BCL6 expression and the Tfh program (46), and tocilizumab treatment decreased the frequency of CXCR5^+^PD-1^hi^ Tfh cells *in vivo*. Second, our data highlight the pleiotropic character and effects of the mbIL-6R signaling pathway on T helper cell fates by inducing the expression of additional transcription factors that have been shown to positively regulate the Th17 (RORγt, BATF) (47–49) or Tfh cell fate (BATF and IKAROS) (50). Importantly, reciprocal expression of BATF and RORγt with IKAROS suggests that different CD4^+^ T cell subsets uniquely sense and respond to IL-6 signaling, which in turn might promote unique effector characteristics or drive the plasticity of Th subsets. The increase in the Th1 specifying transcription factor T-bet and the Th1 chemokine receptor CXCR3 by IL-6 was unexpected but supports prior findings of increased IFN-γ secretion by *in vitro* differentiated Th1 cells following IL-6 pre-exposure (38) and could indicate a role for IL-6 in promoting pathogenic Th1-like Th17 cells in autoimmunity (51). These findings were recapitulated in our *in vitro* differentiation experiments in the presence of TCR stimulation, which further confirmed that, although IL-6 is dispensable in the differentiation of human Tfh cells *in vitro* (11, 41), the presence of IL-6 can enhance features of multiple T helper programs. This is of particular interest as we found an increased frequency of Tfh1/17 cells in carriers of the “risk” IL-6R coding variant rs2228145. Altogether these findings provide new insight into how dysregulated IL-6 responses may drive pathogenic CD4^+^ T helper cell subsets.

Our observation that IL-6 stimulation induced downregulation of TIGIT and CD39 on CD4^+^CD127^lo^CD25^hi^ Treg cells was a novel finding. Previous studies report that TIGIT^+^ Tregs selectively suppress Th1 and Th17 responses (52) and TIGIT signaling counteracts IFN-γ production in Th1 Treg cells which are dysregulated in multiple sclerosis (53). Low expression of TIGIT is also observed on a highly suppressive subset of circulating IL6R^hi^ Tregs at steady state. These harbor a distinct Th17 transcriptome program, including expression of RORγt and IL-17 and may be more susceptible to convert into pathogenic exTreg cells following chronic exposure to a pro-inflammatory environment, which often includes high IL-6 levels (54). Similarly, CD39^+^ Tregs efficiently suppress Th17 responses, are dysregulated in rheumatoid arthritis and multiple sclerosis (55, 56) and increase in rheumatoid arthritis patients responsive to methotrexate (57) and following IL-6R blockade with tocilizumab (55, 58). Our data thus indicate how IL-6 signaling may destabilize the Treg compartment and potentially these markers could be useful to track efficacy of therapies in autoimmunity.

Our findings implicate mbIL-6R signaling in the regulation of FAS-mediated apoptosis, the mechanism through which AICD maintains self-tolerance in terminally differentiated memory and activated T cell subsets (59). We observed a marked increase in FAS expression, which was most pronounced in CD4^+^ TCM cells following IL-6 stimulation. Treatment with tocilizumab reduced the frequency of CCR7^+^CD95^+^ TCM cells, suggesting a novel role for IL-6 signaling in maintaining FAS expression, most evidently on TCM cells. Based on these observations, our finding of higher FAS expression on IL-6R^+^ TEM cells in carriers of the “non-risk/CC” IL-6R coding variant rs2228145 was unanticipated but could be a consequence of altered responsiveness towards alternative IL-6 signaling pathways during T cell activation and differentiation of the non-risk/CC genotype. We provide evidence that a small subset of CD4^+^IL-6R^+^ TEM cells from carriers of the “non-risk/CC’ genotype show higher sensitivity to FAS agonist-induced expression of early apoptotic markers (Annexin V^+^ cells), potentially leading to decreased survival of pathogenic T cells in autoimmunity. Hence, this data provides one potential mechanism for the association of the non-risk allele/CC with protection for development of autoimmunity.

There were a number of limitations to our study. We used primary human T cells for these studies. The use of human samples introduces heterogeneity due to genetic variation, age, sex as well as environmental factors, and the isolation of PBMCs from blood also has the potential to influence cell markers and response to stimuli. To minimize the impact of these challenges, we controlled for age, sex, and the *IL6R* genetic variant known to influence IL-6 signaling, and all samples were processed by a single core laboratory. Our studies were limited to the impact of IL-6 alone, however T cells are not typically exposed to IL-6 in isolation. We chose to do this as a starting point, but future studies studying IL-6 in combination with other forms of activation, such as TCR stimulation or additional cytokines, will be important to expand our understanding of IL-6 and human T cell responses. Moreover, we chose to only use a single dose of a high IL-6 concentration for stimulation in order to ensure the robust phosphorylation of STAT1 and STAT3 in response to mbIL-6R signaling. We also acknowledge that lower availability of IL-6 in the *in vitro* assays may reveal different relationships between IL-6-induced STAT responses and observed T cell phenotypes. Additionally, much of our study is solely focused on classical IL-6 signaling and expression of mbIL-6R but we recognize that trans-signaling through sIL-6R also influences T cell development and function *in vivo*. Due to the use of primary human cells, our ability to prove mechanistic relationships between the pSTAT1:pSTAT3 ratio and outcome is limited, however, the consistency of our findings across platforms, *ex vivo*, *in vitro*, and *in vivo* assessments, supports the proposed relationship with IL-6 signaling.

In summary, we show that the impact of IL-6 on T cells is influenced by the level of mbIL-6R and gp130 expression and this differs with stage of maturation. Our findings indicate that the most prominent pathways influenced by direct IL-6 signaling include differentiation toward Tfh lineage and survival as regulated through the FAS pathway. This also highlights the importance of context with respect to IL-6 signaling – whether alterations occur in naïve cells or committed effector populations. Notably, differences observed were recapitulated in the context of the *IL6R* variant and *in vivo* blockade of IL-6R with tocilizumab demonstrating that alterations in IL6R expression have biological significance *in vivo* on human T cell biology. Collectively, these findings should be taken into account as we consider the role of IL-6 in disease pathogenesis and therapeutic interventions targeting the IL-6 pathway. Our study indicates that the impact of these interventions on T cells will differ with the therapeutic target and stage of T cell maturation. This knowledge will assist in understanding when and how to best target the IL-6 signaling pathway in disease and will provide insight into the adverse effects that may occur from targeting this pathway.

## Data Availability Statement

The datasets presented in this study can be found at https://www.ncbi.nlm.nih.gov/geo/query/acc.cgi?acc=GSE201180. The name of the repository and accession number can be found below: NCBI Gene Expression Omnibus; GSE201180.

## Ethics Statement

The studies involving human participants were reviewed and approved by Benaroya Research Institute Institutional Review Board (IRB7109-471.03). The patients/participants provided their written informed consent to participate in this study.

## Author Contributions

Conceptualization: KL, CH, and JB; Methodology: KL, KD, BJ, CH, and MM; Investigation: KL, KD, BJ, CH, and MM; Analysis: KL, KD, BJ, CH, MM, PL, KC, and JB; Resources: SS, CG, and JB; Writing – Original Draft: KL, AH, and JB; Writing – Review and Editing: KL, KD, BJ, CH, AH, SS, PL, KC, and JB; Visualization: KL, KD, BJ, and CH; Supervision: PL, KC, and JB; Funding acquisition: JB. All authors contributed to the article and approved the submitted version.

## Funding

This work was supported by National Institutes of Health (NIH) grant R01 AI132774 to JB.

## Conflict of Interest

JB is a Scientific Co-Founder and Scientific Advisory Board member of GentiBio, a consultant for Bristol-Myers Squibb and Hotspot Therapeutics, and has past and current research projects sponsored by Amgen, Bristol-Myers Squib, Janssen, Novo Nordisk, and Pfizer. She is a member of the Type 1 Diabetes TrialNet Study Group, a partner of the Allen Institute for Immunology, and a member of the Scientific Advisory Boards for the La Jolla Institute for Allergy and Immunology and BMS Immunology.

The remaining authors declare that the research was conducted in the absence of any commercial or financial relationships that could be construed as a potential conflict of interest.

## Publisher’s Note

All claims expressed in this article are solely those of the authors and do not necessarily represent those of their affiliated organizations, or those of the publisher, the editors and the reviewers. Any product that may be evaluated in this article, or claim that may be made by its manufacturer, is not guaranteed or endorsed by the publisher.
