# Supplementary material for: IL-6-Driven pSTAT1 Response Is Linked to T Cell Features Implicated in Early Immune Dysregulation
Source: Front Immunol. 2022 Jul 13;13:935394. doi: 10.3389/fimmu.2022.935394 (PMC9327741; doi:10.3389/fimmu.2022.935394)
Supplement: Supplementary file 1 [file DataSheet_1.pdf]

Supplemental Figure 1

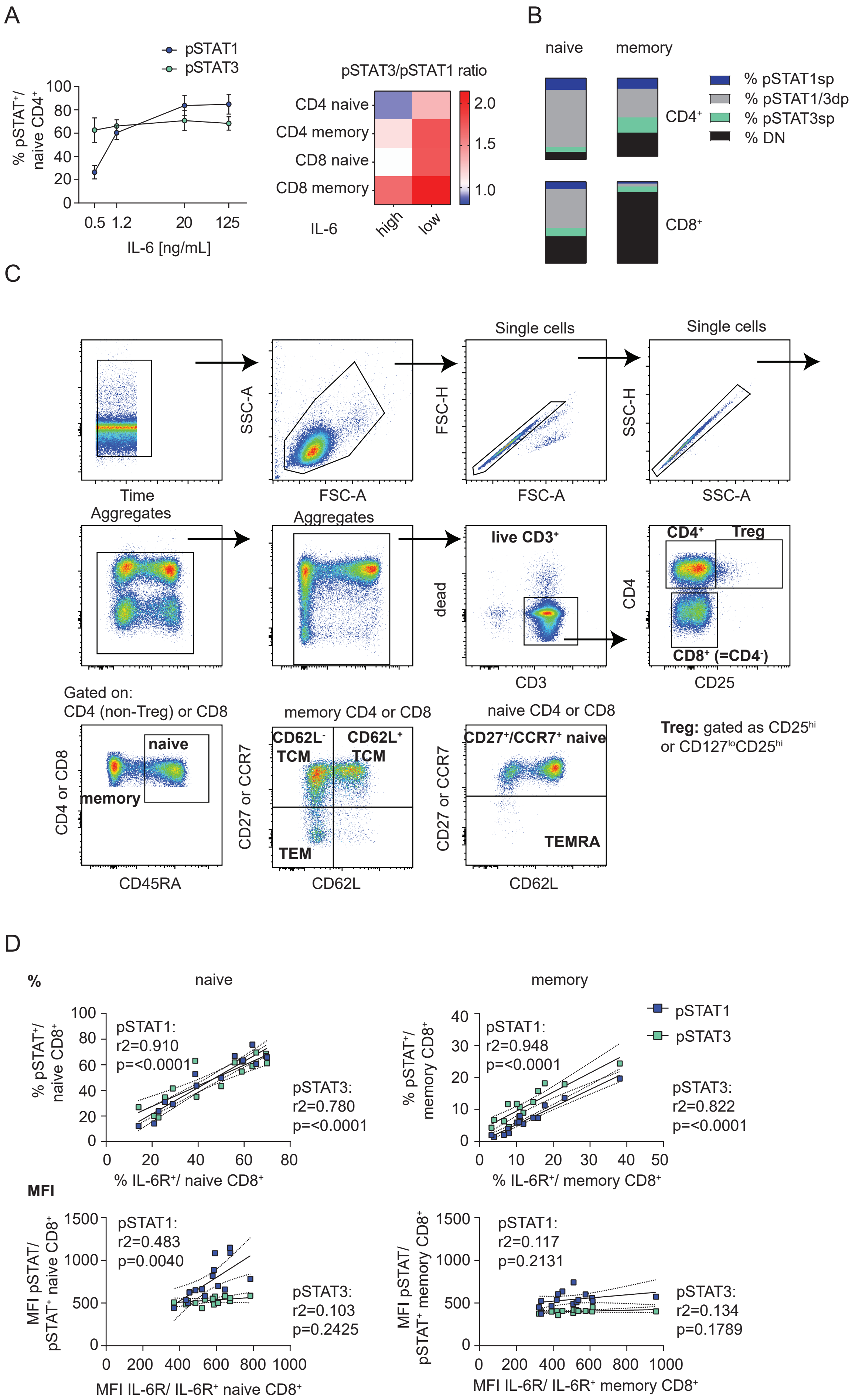

Supplemental Figure 1: IL-6 dose and mbIL-6R expression levels determine pSTAT response in $CD4^+$ and $CD8^+$ T cell subsets. (**A** to **C**) CD3-enriched T cells were stimulated for 30 min with IL-6 and the pSTAT1 and pSTAT3 response was determined by flow cytometry. (**A**)(Left) IL-6 dose titration and (right) balance of phosphorylated STAT proteins in T cells stimulated with a high (20ng/mL) or low (1ng/mL) dose of IL-6. (**B**) Distribution of pSTAT1 and pSTAT3 single positive (sp), double positive (dp) or double negative (DN) T cells among total naive or memory $CD4^+$ or $CD8^+$ T cells. (**C**) Representative gating strategy to define T cell subsets across flow panels. For simplicity, gated $CD3^+CD4^-$ T cells were defined as $CD8^+$ T cells. CD27 and CCR7 were used in different panels as equal markers to define subsets. (**D**) Simple linear regression analysis of expression levels as frequencies (%, top) or MFI (bottom) of gated $mbIL\text{-}6R^+$ and $pSTAT1^+$ or $pSTAT3^+$ cells in total naive or memory $CD8^+$ T cells. Linear regression analysis with Pearson's correlation coefficients. n=15.

Supplemental Figure 2

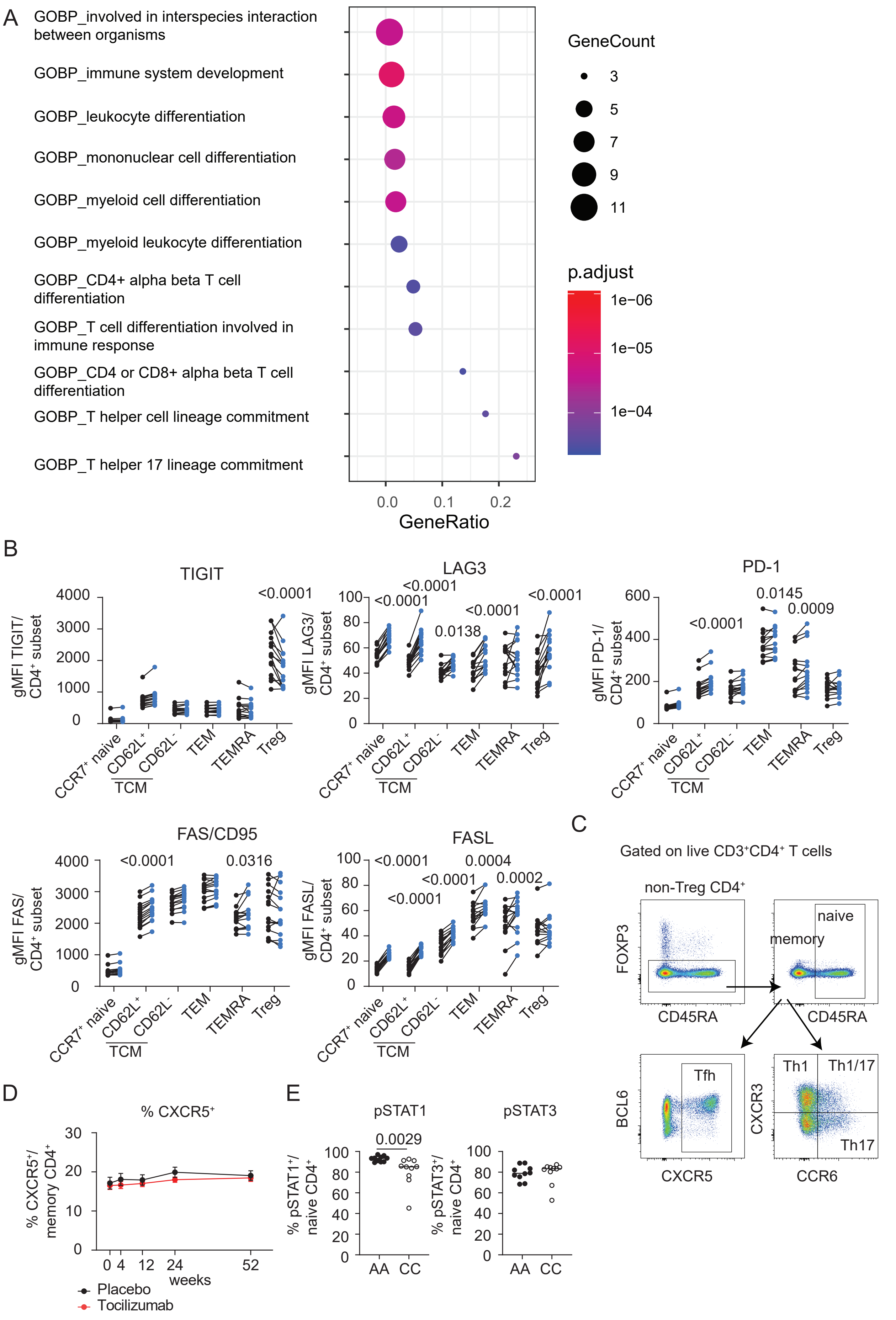

Supplemental Figure 2: (A) RNA-seq analysis reveals IL-6 regulated pathways in T cell subsets. Gene set enrichment analysis using the gene ontology biological processes collection (GOBP) was performed on IL-6 regulated genes common to $CD4^+$ naive, memory and $CD8^+$ naive T cell subsets following 4h of IL-6 stimulation (20 ng/mL) compared to the unstimulated control. (**B**) Increased mbIL-6R signaling drives T cell subset specific phenotypic changes consistent with features observed in autoimmunity. CD3-enriched T cells were stimulated with IL-6 (20 ng/mL, circles in blue) or left unstimulated (circles in black) for 24h and the change in gMFI expression levels of selected surface markers in gated $CD4^+$ T cell subsets was determined via flow cytometry. n=15. 2-Way ANOVA and Bonferroni's multiple comparisons test. **(C)** Representative gating strategy to define Th cell subsets. **(D)** Frequencies of $CXCR5^+$ memory CD4+ (non-Treg) T cells over time in the tocilizumab treated versus placebo group. (**E**) $CD4^+$ T cells from carriers of the mbIL-6R rs2228145 SNP "risk" (AA) genotype show increased pSTAT1 response following IL-6 stimulation in their $CD4^+$ naive compartment.
